# HIV preventive behavior and associated factors among mining workers in Sali traditional gold mining site bench maji zone, Southwest Ethiopia: a cross sectional study

**DOI:** 10.1186/1471-2458-14-1003

**Published:** 2014-09-26

**Authors:** Hordofa Gutema Abdissa, Yohannes Kebede Lemu, Dejene Tilahun Nigussie

**Affiliations:** Mizan-Aman Hospital, Bench Maji Zonal health facility, Mizan, Ethiopia; Department of Health Education & Behavioral Sciences, Jimma University, Jimma, Ethioipia

**Keywords:** HIV preventive behavior, Health belief model, Traditional gold mining, Ethiopia

## Abstract

**Background:**

Prevalence of HIV and other STI is high among migrant mining workers due to factors such as dangerous working conditions, only masculine identities existence, living away from families, desolate and in hospitable place. This makes them known to be HIV and STI vulnerable group in different part of the world. But, in Ethiopia they were not thought as at risk group yet. So the aim of this study is to assess magnitude of HIV preventive behaviours and associated factors among gold miners in Sali traditional gold mining site.

**Methods:**

A cross sectional study was conducted to assess HIV preventive behavior of the mining worker. The data were collected using interviewer administered structured questionnaire adapted from other related behavioural studies. The data was entered using EPI data version 3.1and analyzed using SPSS version 17. Multiple logistic regression was used to assess relationship of HIV preventive behavior with constructs of health belief model.

**Results:**

A total of 393 respondents with response rate of 93.12% were participated. All of the study participants were male 393(100%), the mean age of the participant was 24.0 (±5.13SD). Less than half of the respondents 187(47.6%) were engaged in HIV preventive behavior. Less than half (45.3%) of them have high perceived susceptibility to HIV/AIDS; majority (62.8%) of them has high perceived severity to HIV/AIDS. HIV preventive behavior is negatively associated with being in middle, higher and highest income [OR = 0.54, 95% CI: 0.21, 0.74], [OR = 0.40, 95% CI: 0.30, 0.98] and [OR = 0.39, 95% CI: 0.20, 0.77] respectively and positively associated with Completing secondary, tertiary school and self efficacy [OR = 2.66, 95% CI: 1.11, 6.41], [OR = 5.40, 95% CI: 1.54, 19] and [OR = 1.88, 95% CI: 1.18, 2.94] respectively.

**Conclusions:**

The HIV preventive behavior of the mining worker was low. Being engaged in sexual intercourse with one sexual partner is very low, Consistent condom use among these mining workers was low. Income, educational status and self efficacy have significant effect on the HIV preventive behavior of mining workers. Thus this population group should be understood as at risk population at national level.

## Background

Infection with HIV constitutes a major health crisis worldwide. Currently the number of people living with HIV/AIDS in the world is estimated 34 million. This large number is due to significant expansion of access to antiretroviral therapy and reduced AIDS related death. But still every day approximately 7,100 new infections of HIV and 4,900 AIDS related deaths occur worldwide. Of the estimated 34 million people infected with HIV worldwide in 2010, sub-Saharan Africa accommodated 68% of this number. In Ethiopia being infected HIV is declining, but the number of people living with the HIV virus in the country is still large. The total number of people living with HIV in the country is more than 1.3 -1.5 million and the estimated prevalence rate is between 1.4% and 2.8% [1].

The country faces HIV epidemic among different sub-populations and geographic areas. Population segment such as uniformed services, sex workers, long distance truck drivers, refugees and displaced peoples, daily laborers, migrant laborers, steer children, high school and university students, out of school youth and indigenous population are known to be most at risk population. But mining workers were not constructed as at risk population group since there is lack of sufficient evidence on the HIV among this population group in the country [2, 3]. Prevalence of HIV and other STI is high among mining workers due to factors such as dangerous working conditions, only masculine identities existence, living away from families, limited access to healthcare and settling in isolated, desolate and in hospitable place. This makes them known to be HIV and STI vulnerable group in different part of the world [4–6].

Nowadays, the impact of HIV/AIDS is the forefront factor that constrains the economic development of the country especially the low income developing countries. Evidences showed that consistent condom use and having one sexual partner among mining worker was low and their visiting of commercial sex worker was high and constructing this population group known to be highly at risk group to HIV infection in different part of the world [4, 7–9].

Health Belief Model (HBM) has been used most widely as conceptual frameworks in health sexual behavior research in relation to abstaining, condom use, having steady sexual partner and being tested for HIV infection [10–12]. According to the Health Belief Model, individuals are more likely to adopt safer sexual behaviors if they perceive that:- they are susceptible to HIV/AIDS infection (perceived susceptibility); the consequences of HIV/AIDS infection are serious (perceived severity); abstaining from sex, condom use, a single sexual partner or being tested for HIV infection are beneficial to HIV/AIDS prevention (perceived benefits); barriers to abstaining from sex, condom use, have single sexual partner or testing for HIV infection (perceived barriers) are low and are confident in ability to abstain, use condom, have steady sexual partner and tested for HIV(self efficacy) [13]. These five perceptions are important determinants of abstaining, condom use, having steady sexual partner and testing for HIV infection. Perceived susceptibility and perceived severity were significantly associated with abstinence, condom use and testing for HIV infection [10–12, 14, 15]. Perceived barrier and self efficacy were also significantly related with these prevent behaviors [12, 16, 17]. But perceived benefit did not found to be predictor of any of these health behaviors in various studies.

Sali traditional gold mining site is among gold mining site in the country which is found in bench maji zone southwest of country. The populations in the area were not permanent settler. Since they stay in one site for sometime where they assume they can get more gold. They leave that site and go to another place where they assume they can get more gold. Almost all of the miners are young age group migrants who migrate from different part of the country to get better income from the mining. The main income source for the mining workers in the site is from the gold they sell at site. While for other population group in the site commercial sex work, selling of alcohol are the main source of income. However, it is not yet identified the level of their being engaged in HIV prevention and whether the constructs of health belief model are related to their HIV preventive behavior. It was hypothesized that mining workers with higher perceptions would engaged in HIV preventive behavior. Hence, this study examined the HIV preventive behavior of the mining worker and the association between HBM constructs and HIV preventive behavior.

## Method

### Study design and population

This study used cross-sectional design and conducted among mining workers in Sali traditional gold mining site southwest Ethiopia. It is estimated that 12,000 people living in the site. There is only one health post and ten private clinics serving the mining site population and no any HIV prevention program yet implemented in the site. Additionally, there is no communication, road facility and school in the site yet.

### Participants and sampling

The source populations were all gold miners in the site and study populations were all sampled gold miners working in site and who were happen to be present during study period. Individuals who stayed in the site for more than three month were included. While who were unable to communicate due to severe illness or deafness excluded from the study.

The sample size was calculated assuming proportion of 50% for consistent condom use, confidence interval of 95% and 5% margin of error (d). The final sample size was determined to be 422 after adding 10% for possible non-response.

The sampling frame was prepared with respective of their small site where they specifically work. Then using the simple random sampling technique the individual to be involved in the study was selected.

### Operational definitions

**HIV preventive behavior-** if an individual is abstaining from sexual intercourse in the last one year until the time of study period, using condom consistently or have only one partner in the past one year, having only one sexual partner and tested for HIV before their first sexual relation in the last six month, tested for HIV infection in last three month of the study period and consistently use condom it was said to be in HIV preventive behavior.

### Data collection procedure

The study was conducted from Jan 15, 2012 to Feb 15, 2012. The questionnaire was initially prepared in English and then translated into Amharic. The Amharic version was again retranslated back to English by other individual to check for consistency of meaning. The translated Amharic version questionnaire was pre-tested prior to the actual data collection on 5% of the sample size outside of the study area from similar population. The trained six data collectors who had completed 10 to 12 grades and two supervisors who were first degree holder health profession were recruited. Those randomly selected individual was found by arranging with site leaders to know their respective small site where they specifically work. Finally the data was collected at small site where they were working.

### Instruments

The data were collected using interviewer administered structured questionnaire adapted from other related behavioural studies [10, 15, 16, 18]. It comprised of socio demography characteristics, HIV preventive behaviour (9 items), perceived susceptibility (6 items), perceived severity (6 items), perceived benefit (16 items), perceived barrier (16 items), self efficacy (16 items) and cue to preventive action (12 items). Every constructs of HBM items response were elicited using five-point likert scale: strongly disagree (1),” “disagree (2),” “neither agree nor disagree (3),” “agree (4),” “strongly agree (5).” Negatively worded items were reversely coded during analysis. Reliability of each items of HBM constructs were checked using cronbach’s alpha (α). After dropping one item, the alpha of perceived susceptibility was α = 0.73, perceived severity α = 0.71 having all items, perceived benefit α = 0.87 without dropping any item, perceived barrier α = 0.75 after dropping four items, self efficacy α = 0.84 after dropping one item and cue to preventive action α = 0.82 by including all items. Finally the sum of each items remained in each scale were used as a construct of HBM.

### Statistical analysis

The data was entered using EPI data version 3.1 and analyzed using SPSS Statistics software version 17.0. Data cleaning and assumption checking were performed prior to proceeding to analysis. Descriptive statistical analysis was done, T-test and chi square was used as needed to check the association between HIV preventive behavior, constructs of HBM and modifying factors (socio demographic characteristics). Multiple logistic regression analysis was used for prediction of HIV preventive behavior in three different models. In the first regression model, the effect of the socio demographic characteristics was assessed. While in the second model the constructs of HBM was assessed, in the final model those independent variables which had statistical significant association with the outcome variable in the two model above were entered the final regression model. To claim statistically significant effect, crude and adjusted odds ratio with 95% confidence interval (CI) was employed.

### Ethical consideration

The study protocol was approved by the Ethical Clearance Committee of Jimma University and written informed consent was sought from each respondent before the interview. The data obtained in due course were confidentially stored.

## Result

A total of 393 respondents participated in this study producing a response rate of 93.12%.

### Socio demographic characteristics

Table 1 shows the socio demographic characteristics of the respondents. Accordingly, all of the study participants were male 393 (100%), the mean age of the participant was 24.0 (±5.13SD). One hundred eighty two (46%) of the respondents were orthodox in religion. Concerning ethnicity, large number (42.5%) of the respondents were Amhara in their ethnicity, followed by Oromo (15%). Around 7 in 10 (69.0%) of the participants were single. Almost half (49.1%) of them completed secondary school. Regarding the monthly income large number (29.8%) of the respondents were in higher income quartile.

**Table 1:** **Socio demographic characteristics of mining workers in Sali traditional gold mining site Bench Maji Zone South West Ethiopia, February 2012 n = 393**

Variables	Frequency	Percent
**Age**		
**15-19**	53	13.5
**20-24**	185	47.1
**25-34**	134	34.1
**35 and above**	21	5.3
**Religion**		
**Orthodox**	182	46.3
**Muslim**	108	27.5
**Protestant**	88	22.4
**Others**	15	3.8
**Ethnic group**		
**Amhara**	167	42.5
**Oromo**	59	15.0
**Walayita**	53	13.5
**Hadiya**	51	13.0
**Gurage**	46	11.7
**Others**	17	4.3
**Marital status**		
**Single**	271	69.0
**Married**	105	26.7
**Divorced**	32	2.5
**widowed**	26	1.8
**Monthly income** ^*^		
**Lower**	99	25.2
**Middle**	98	24.9
**Higher**	117	29.8
**Highest**	79	20.1
**Educational status**		
**can’t read and write**	35	8.9
**Primary school**	142	36.1
**Secondary school**	193	49.1
**Tertiary**	23	5.9

*Income categorized using quartile.

### HIV preventive behaviors

As shown on the Table 2 the HIV preventive behavior and risky behavior to HIV risk was presented. Less than one fourth (23.5%) of them have only one sexual partner in the last twelve months. One hundred seventy (43.5%) of the respondents were undergone HIV testing in the last three of the study period. Of those who have more than one sexual partner only small number (35.5%) of them were using condoms consistently. Around half (47.8%) of them engaged in sex with commercial sex workers. Out of those who are sexually active and use condom, less than half (46%) of them were using condom consistently in the last twelve months. From those who visited commercial sex workers and used condom only 56(41.48%) of them used condom consistently. Above half (52.4%) of them were not engaged in HIV preventive behavior which was obtained by counting from each component.

**Table 2:** **Frequency distribution of HIV preventive behavior among mining workers in Sali traditional gold mining site Bench Maji Zone, South West Ethiopia, Feb 2012 N = 393**

Variables	Frequency	Percent
**Currently abstaining from sex**		
**Yes**	31	7.9
**No**	362	92.1
**Consistent condom use**		
**Yes**	127	46
**No**	149	54
**Have one sexual partner**		
**Yes**	86	23.5
**No**	276	76.5
**Undergone HIV testing in the last three month**		
**Yes**	170	43.5
**No**	221	56.5
**With whom engaged in sex**		
**Same sex**	9	2.5
**Commercial sex workers**	173	47.8
** Regular partner**	86	23.7
** Casual partner**	94	26
**HIV preventive behavior**		
** Yes**	187	47.6
** No**	206	52.4

### Socio demographic predictors of HIV preventive behavior

The relationship between socio-demographic variables and HIV preventive behavior is presented in Table 3. In this model only income and educational status were significantly associated with HIV preventive behavior. Those whose monthly income is in middle, higher and highest level were less likely to be engaged in HIV behaviors as compared with those whose monthly income in lower level. Those workers who completed secondary school and tertiary school were more likely engaged in HIV preventive behavior as compared with those who are illiterate.

**Table 3:** **Socio demographic characteristics and their association with HIV preventive behavior among mining workers in Sali traditional gold mining site Bench Maji Zone, South West Ethiopia, Feb 2012 N = 393**

Variables	HIV preventive behavior		OR (95% CI)	
	Yes	No	Crude	Adjusted
	n (%)	n (%)		
**Age of respondent**				
**15-19**	21(5.3%)	32(8.1%)	1(reference)	1(reference)
**20-24**	93(23.7%)	92(23.4%)	1.54(0.82, 2.86)	1.51(0.79, 2.91)
**25-34**	67(17.0%)	67(17.0%)	1.52(0.80, 2.86)	1.60(0.81, 3.17)
**35 and above**	6(1.5%)	15(3.8%)	0.61(0.20, 1.82)	0.56(0.17, 1.82)
**Religion**				
**Orthodox**	92(23.4%)	90(22.9%)	1(reference)	1(reference)
**Muslim**	46(11.7%)	62(15.8%)	0.72(0.44, 1.17)	0.76(0.45, 1.30)
**Protestant**	44(11.2%)	44(11.2%)	0.97(0.588,1.62)	1.01(0.56,1.844)
**Others**	5(1.3%)	10(2.5%)	0.48(0.16, 1.48)	0.65(0.12, 2.16)
**Ethnic status**				
**Amhara**	74(18.8%)	93(23.7%)	1(reference)	1(reference)
**Oromo**	30(7.6%)	29(7.4%)	1.30(0.71, 2.35)	1.34(0.68, 2.63)
**Gurage**	26(6.6%)	20(5.1%)	1.63(0.84, 3.15)	1.47(0.72, 3.00)
**Walayita**	25(6.4%)	28(7.1%)	1.12(0.64, 2.08)	100(0.47, 2.11)
**Hadiya**	21(5.3%)	30(7.6%)	0.88(0.46, 1.66)	0.99(0.48, 2.06)
**Others**	11(2.8%)	6(1.5%)	2.30(0.81, 6.52)	2.18(0.73, 6.52)
**Marital status**				
**Single**	131(33.3%)	140(35.6%)	1(reference)	1(reference)
**Married**	50(12.7%)	55(14.0%)	0.98(0.61, 1.52)	0.90(0.55, 1.49)
**Divorced**	3(0.8%)	7(1.8%)	0.45(0.11, 1.80)	0.55(0.13, 2.30)
**Widowed**	3(0.8%)	4(1.0%)	0.80(0.17, 3.64)	0.94(0.19, 4.50)
**Monthly income**				
**Lower**	60(15.3%)	39(9.9%)	1(reference)	1(reference)
**Middle**	42(10.7%)	56(14.2%)	0.48(0.28, 0.86)*	0.41(0.22, 0.76)*
**Higher**	56(14.2%)	61(15.5%)	0.59(0.34, 1.02)	0.53(0.23, 0.94)*
**Highest**	29(7.4%)	50(12.7%)	0.38(0.20, 0.70)*	0.36(0.18, 0.69)*
**Educational status**				
**Illiterate**	10(2.5%)	25(6.4%)	1(reference)	1(reference)
**Primary school**	63(16.0%)	79(20.1%)	2.00(0.89, 4.45)	2.21(0.94, 5.21)
**Secondary school**	98(24.9%)	95(24.2%)	2.57(1.17, 5.65)*	3.00(1.28, 6.82)*
**Tertiary**	16(4.1%)	7(1.8%)	5.71(1.8, 18.08)*	5.27(1.55, 17.9)*

*Significant at p < 0.05.

### HBM constructs as predictors of HIV preventive behavior

The effect of HBM constructs on HIV preventive behavior is showed on Table 4. From this model only self efficacy was significantly associated with HIV preventive behavior. But perceived benefit was significantly associated with HIV preventive behavior on Bivariate analysis. Those who have high perceived benefit were more likely to be engaged in HIV preventive behavior when compared with those who were not in HIV preventive behavior. The likelihood of being engaged in HIV preventive behavior was higher among those who had high self efficacy on preventing HIV HIV/AIDS as compared with those who had low self efficacy on preventing HIV/AIDS.

**Table 4:** **Perception toward HIV/AIDS and their association with HIV preventive behavior of mining workers in Sali traditional gold mining site Bench Maji Zone, South West Ethiopia, Feb 2012 N = 393**

Perceptions	HIV preventive behavior		OR (95%CI)	
	Yes	No	Crude	Adjusted
	N (%)	N (%)		
**Perceived susceptibility**				
High*	80(20.4%)	98(24.9%)	0.82(0.55, 1.22)	0.82(0.54, 1.25)
Low**	107(27.2%)	108(27.5%)	1.00(reference)	1.00(reference)
**Perceived severity**				
High*	118(30%)	129(32.8%)	1.02(0.67, 1.53)	0.94(0.61, 1.44)
Low**	69(17.6%)	77(19.6%)	1.00(reference)	1.00(reference)
**Perceived benefit**				
High*	117(29.8%)	106(27%)	1.57(1.05, 2.36)***	1.43(0.93, 2.21)
Low**	70(17.8%)	100(25.4%)	1.00(reference)	1.00(reference)
**Perceived barrier**				
High*	100(25.4%)	108(27.5%)	1.04(0.70, 1.55)	1.12(0.74, 1.70)
Low**	87(22.1%)	98(24.9%)	1.00(reference)	1.00(reference)
**Self efficacy**				
High*	117(29.8%)	95(24.2%)	1.95(1.30, 2.92)***	1. 86(1.21, 2.86)***
Low**	70(17.8%)	111(28.2%)	1.00(reference)	1.00(reference)
**Cue to action**				
High*	121(30.8%)	120(30.5%)	1.31(0.87, 1.97)	1.09(0.83, 1.44)
Low**	66(16.8%)	86(21.9%)	1.00(reference)	1.00(reference)

*Above mean, **Below mean, ***Significant at p < 0.05.

### Independent predictors of HIV preventive behavior

Variables which had statistically significant association with HIV preventive behavior in the above two models were entered into the final model. As shown in Table 5, the three variables which were significant in the two models were still significant in the final model. Thus monthly income, educational status and self efficacy were remained to be significant predictor of HIV preventive behavior in the final model. Being engaged in HIV preventive behavior was 46%, 60% and 61% less likely among mining workers whose monthly income found in middle, higher and highest level as compared with those whose monthly income found in lower level [OR = 0.54, 95% CI: 0.30, 0.98], [OR = 0.40, 95% CI: 0.21, 0.74], and [OR = 0.39, 95% CI: 0.20, 0.77] respectively. Mining Workers who completed secondary school and tertiary school were 2.66 and 5.4 times more likely to be engaged in HIV preventive behavior as compared with those who were illiterate [OR = 2.66, 95% CI: 1.11, 6.41) and [OR = 5.40, 95% CI: 1.54, 19] respectively. The likelihood of being engaged in HIV preventive behavior is 1.88 times higher among those having high self efficacy toward HIV prevention as compared with those who have low self efficacy toward HIV prevention [OR = 1.88, 95% CI: 1.18, 2.94].

**Table 5:** **Independent predictors of HIV preventive behavior of mining workers in Sali traditional gold mining site Bench Maji Zone, South West Ethiopia, Feb 2012 N = 393**

Variables	HIV preventive behavior		OR (95% CI)	
	Yes	No	Crude	adjusted
	N (%)	N (%)		
**Monthly income**				
**Lower**	60(15.3%)	39(9.9%)	1(reference)	**1**(reference)
**Middle**	42(10.7%)	56(14.2%)	0.48(0.28, 0.86)*	0.54(0.30, 0.98)*
**Higher**	56(14.2%)	61(15.5%)	0.59(0.34, 1.02)	0.40(0.21, 0.74)*
**Highest**	29(7.4%)	50(12.7%)	0.38(0.20, 0.70)*	0.39(0.20, 0.77)*
**Educational status**				
**Illiterate**	10(2.5%)	25(6.4%)	1(reference)	1(reference)
**Primary school**	63(16.0%)	79(20.1%)	2.00(0.89, 4.45)	2.07(0.85, 5.05)
**Secondary school**	98(24.9%)	95(24.2%)	2.57(1.17, 5.65)*	2.66(1.11, 6.41)*
**Tertiary**	16(4.1%)	7(1.8%)	5.71(1.8, 18.08)*	5.40(1.54, 19.00)*
**Perceived benefit**				
**High**	117(29.8%)	106 (27%)	1.57(1.05, 2.36)*	1.42(0.88, 2.31)
**Low**	70(17.8%)	100 (25.4%)	**1**(reference)	1(reference)
**Self efficacy**				
**High**	117(29.8%)	95(24.2%)	1.95(1.30, 2.92)*	1.88(1.18, 2.94)*
**Low**	70(17.8%)	111(28.2%)	**1**(reference)	**1**(reference)

*Significant at p < 0.05.

## Discussion

This study gives important information regarding the sexual behavior of mining workers and their perceptions toward HIV/AIDS. In this study, Out of those sexually active respondents only (23.5%) of them have only one sexual partner in the last twelve month of study time. This figure is lower than previously reported findings among mining workers [6, 19]. Out of those who were sexually active and use condom (46%) of them were using condom consistently in the last twelve months. This finding is higher when compared with other studies finding [4, 8, 9]. The difference might be due to some knowledge gap on the importance of condom use among the mining workers in those of studies. In this study 43.5% of mining workers were under gone testing for HIV in the last three of the study period. This is almost the same to study conducted previously [14, 15].

Mining workers who completed secondary school and tertiary school were 2.66 and 5.4 times more likely engaged in HIV preventive behavior respectively as compared with those who were illiterate. Previous studies also support this finding [14, 15]. Mining workers whose monthly income is middle, higher and highest were less likely to be engaged in HIV preventive behaviors as compared with those whose monthly income is lower by 60%, 46% and 61% respectively. This finding is also similar with one study conducted in Thailand [20]. Perceived Self efficacy was the only construct from HBM constructs which showed independent significant relation with HIV preventive behavior in this study. Mining workers who had high perceived self efficacy were 1.88 times more likely to practice HIV preventive behavior as compared with those who had low perceived self efficacy. This was also the same with different studies done in different areas. In study conducted in America among Taiwanese immigrant only self efficacy was significant predictor of HIV prevention [18]. In generally self efficacy was predictor of HIV preventive behavior in different studies [11, 12, 15, 17]. In this study other constructs of HBM did not show independent association with HIV preventive behavior. But in previous studies they have significant effect on HIV preventive behavior [10–12, 16]. This difference is due to outcome variable which was the combination of different category of preventive behavior in this study while other studies used individual component in their study.

### Limitation of the study

It has to be noted that the data was collected using interviewer administered questionnaire which may make the finding prone to social desirability bias. Since the study was done using cross sectional design, it is difficult to know whether the behavior or the predicting variable occurred first.

## Conclusion

The finding from this study indicated that the HIV preventive behavior of the mining worker was low. Being engaged in sexual intercourse with one sexual partner is very low, Consistent condom use among these mining workers was low. Besides this having sexual intercourse with commercial sex workers was high. Income, educational status and self efficacy have significant effect on the HIV preventive behavior of mining workers. The finding of this study has important implication in understanding this population segment as most at risk population segment in a country like Ethiopia where they were not yet taken as at risk. Interventional program such as community-based outreach which bring behavior change and which make the target group involved as fully as possible in design and implementation of the program should be implemented. It could also direct the researcher toward understanding of behavioral change after interventions among mining workers.
